# Arterial Compression as an Antiphlogistic

**Published:** 1849-09

**Authors:** 


*Arterial Compression as an Antiphlogistic.*—Dr. Henroz de Marche has published a work on the value of compressing the brachial artery in cases of whitlow to check the inflammatory process in the finger; this seems but an exaggeration of M. Gerdy's principle of keeping the limb elevated so as to lessen the force of the arterial circulation in the inflamed part. Dr. Henroz was one day in his garden pruning an arbutus, and got a prick of a thorn in his left ring-finger at the inner side of the third phalanx; the thorn was extracted, and for twenty-four hours he felt no uneasiness in the part; the finger at this time began to swell rapidly, and to grow red, and the inflammation extended by degrees to the palm and back of the hand. On the fourth day, the pain was pulsatile and severe; he could not sleep; had great thirst; skin hot, and pulse frequent; the axillary glands were swollen but indolent. Stuping, leeches, poultices, opiates, mercurial frictions, were in their turn tried without advantage. It then occurred to M. Henroz to try compression of the brachial artery, which he did immediately with his thumb—instantly, the severe pain which he had endured for five days ceased, as if by magic, and he was able, without the slightest uneasiness, to put his hand into any position he pleased, and even the redness disappeared completely. However, as it was impossible to maintain the pressure in this manner for any length of time, he contrived an instrument for the purpose, so simple in its construction as perhaps to make it a valuable aid in such cases in the country, where more perfect ones could not be readily had. It was applied on the brachial artery, and the same good effects immediately followed as when compression was made with the thumb; it was left on for three hours, during which the pain in the hand did not recur for an instant; it was pale and cool, and the swelling had diminished. Fearing that a longer interruption to the circulation might produce ill consequences, M. Henroz suspended the compression for three-quarters of an hour. The pain returned; pressure was again made, but this time it was on the ulnar not the brachial artery, and the symptoms were as suddenly relieved as in the former case.—Compression on the artery was thus continued from half-past twelve at noon until five o'clock in the evening, as well as the palm and dorsum of the hand with firm compresses of wadding, at which time the tumefaction of the hand and finger was permanently reduced, as also the tenderness; the symptoms of reaction had ceased, and there were no longer pain or fever. In the evening, pressure was again made; and continued all night; the next day the cure was complete.

The same treatment was employed by M. Henroz with the same result on a young girl who had a very severe whitlow; in this case, in which the affection was eight days progressing, the pain left the part the instant the compression was applied, and the cure was complete in thirty-six hours.—*Journal de Médicine.*

